# Variation Patterns of the Volatiles during Germination of the Foxtail Millet (*Setaria Italic)*: The Relationship between the Volatiles and Fatty Acids in Model Experiments

**DOI:** 10.3390/molecules25051238

**Published:** 2020-03-09

**Authors:** PengLiang Li, Yin Zhu, ShaoHui Li, AiXia Zhang, Wei Zhao, JiaLi Zhang, QinCao Chen, SuFen Ren, JingKe Liu, HuiJun Wang

**Affiliations:** 1 Institute of millet crops, Hebei Academy of Agriculture and Forestry Sciences, 162 Hengshan Street, Shijiazhuang 050035, China; lpl1937@126.com (P.L.); lishaohui007@163.com (S.L.); zhangaixia1977@126.com (A.Z.); zhaoweipg@163.com (W.Z.); zhangjiali416@126.com (J.Z.); 17736491391@163.com (S.R.); 2 Key Laboratory of Tea Biology and Resources Utilization, Ministry of Agriculture, Tea Research Institute, Chinese Academy of Agricultural Sciences, 9 Meiling South Road, Hangzhou 310008, China; zhuy_scu@tricaas.com (Y.Z.); chenqincao@jxau.edu.cn (Q.C.)

**Keywords:** volatile, fatty acid, variation, germination, foxtail millet

## Abstract

Functional and nutritional compounds are increased during foxtail millet germination while bad smell is produced due to the fatty acid oxidation. To eliminate the unpleasant aroma, the origins of the volatiles must be known. A comprehensive two-dimensional gas chromatography-time-of-flight mass spectrometry showed forty-nine volatiles containing 8 ketones, 10 aldehydes, 20 alkanes, 4 alcohols, 5 alkenes, and 2 furans were tentatively identified, and they increased during the germination of the foxtail millet. To identify the origin of some volatiles, model experiments by adding 6 fatty acids to the crude enzymes of the foxtail millet was designed, and 17 volatiles could be detected. The saturated fatty acids (palmitic acid and stearic acid) had no contributions to the formation of the volatiles, whereas the unsaturated fatty acid played important roles in the formation of volatiles. Among the unsaturated fatty acids, palmitoleic acid and linoleic acid produced most aldehydes, alcohols, and ketones, while linolenic acid produced the most alkanes and alkenes. This study will be helpful for controlling the smell of germinated seeds from the raw material selection.

## 1. Introduction

Seed germination is considered an efficient method for improving the nutritional components of cereals [1,2]. Amino acids, fatty acids, and some vitamins are increased during the germination of *Brassica juncea* [1], foxtail millet [3], and peanut kernel [4]. In addition, functional components such as flavonoids [5,6], phenolic acids [6], and γ-aminobutyric acid (GABA) [7,8] are also improved during seed germination. It shows the germinated seed is a potential source of functional food.

However, smell is produced in germinated seed and the hydroperoxidation of the fatty acids plays important role in its production. A seed contains a high level of lipid for energy storage. During its germination, the lipid is hydrolyzed to free fatty acid by the lipase, and the unsaturated fatty acid is oxidized to hydroperoxide by lipoxygenase (LOX) [4,9]. The hydroperoxide can be degraded to the volatiles of alkanes, aldehydes, ketones, and alcohols by hydroperoxide lyase (HPL) and alcohol oxidoreductase [10,11]. Furthermore, the activities of lipase, LOX, and HPL become higher during seed germination [9,12,13].

Though these volatiles have been detected in many plants and have been known produced by the fatty acid oxidation [14,15], there is hardly any documentary evidence clearly described the correlations between the volatiles and the corresponding fatty acid. Moreover, it is difficult to use a mono-enzyme to contact the volatiles to the individual fatty acid because there are isomers of HPL existed in plants and they have different substrate specificities [10,11]. Thus, using the crude enzyme can conveniently correlate the fatty acid and volatiles. With the crude enzyme extract from leeks, linoleic acid can produce the hexanal, heptanal, (*E*)-2-heptenal, 1-octen-3-ol, (*E*)-2-octenal, (E, *E*)-2,4-decadienal, and pentanol, whereas linolenic acid can produce the hexenal, (*E*)-2-pentenal, (E, *Z*)-2,4-heptadienal, (*E*, *E*)-2,4-heptadienal, butanol, and *(E)*-2-hexenal [10]. However, diverse fatty acids exist in seeds, and diverse volatiles are produced during germination. A comprehensive study that relates volatiles with other individual fatty acid precursor is still needed.

Foxtail millet (*Setaria italica*) is one of the important food crops with high nutrition in China. During its germination, functional components were significantly improved [7]. However, unpleasant smell produced by fatty acids influences consumer acceptance. To eliminate the bad smell, the precursors must be known. The aim of this study is to identify the origins of the volatiles from germinated foxtail millet, and the relationship between the volatiles and the fatty acid was analyzed by a model experiment.

## 2. Results and Discussion

### 2.1. Variations of the Volatiles during the Germination of Foxtail Millet

**Figure 1:**
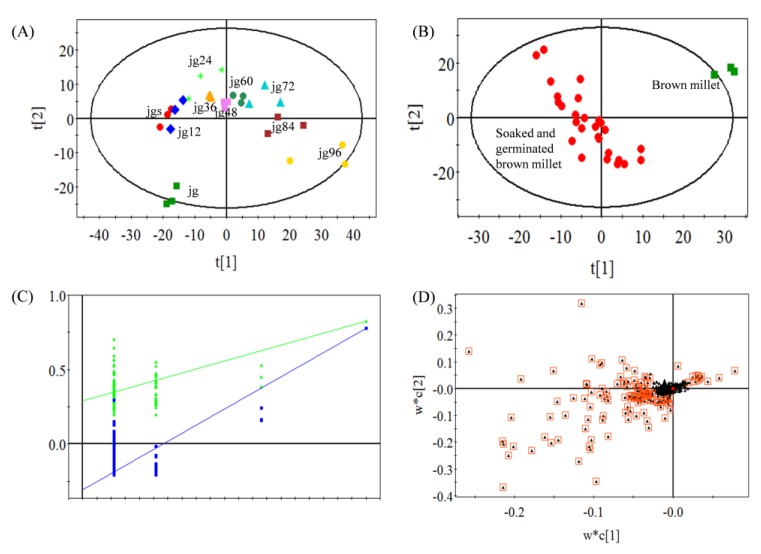
Multivariate statistical analysis of the volatiles during foxtail millet germination: (**A**) principal component analysis (PCA) score plot; (**B**) Partial least squares-discriminate analysis (PLS-DA) score plot, R^2^X = 0.523, R^2^Y = 0.827, Q^2^ = 0.778; (**C**) Cross-validation plot of the PLS-DA model with 100 permutation tests (intercepts of R^2^ and Q^2^ are 0.306 and −0.268, respectively); (**D**) PLS-DA loading plot (black triangles with boxes represented most differential metabolites). jg, jgs, and jg12–96 represented the raw brown millet, soaked brown millet, and germinated millet, respectively.

To comprehensively understand the variation of volatiles during millet germination, a comprehensive two-dimensional gas chromatography-time-of-flight mass spectrometry (GC×GC-TOF/MS) was applied to detect the volatiles in the samples of brown millet, soaked millet, and germinated millet. After the peak alignment, a total of 449 ions were obtained for statistical analysis. A principal component analysis (PCA) divided the samples into two groups (Figure 1A), and the first two principal components explained 40.6% of the total variance (29.6% and 11.0%, respectively). The group contained jg (raw brown millet) was below the group contained jgs, jg12, jg24, jg36, jg48, jg60, jg72, jg84, and jg96 (jgs and jg12–96 represented the soaked brown millet and germinated millet for 12–96 h, respectively), and the samples in the latter group aligned from the left to right. This indicated that some different volatiles were produced during soaking and germination of the foxtail millet. A supervised partial least squares-discriminate analysis (PLS-DA) was then performed in the two groups (Figure 1B), and the model was no overfitting by cross-validation (Figure 1C). The loading plot of the PLS-DA model was used to screen the main differential volatiles in the two groups (Figure 1D). Finally, 49 volatiles which contained 8 ketones, 10 aldehydes, 20 alkanes, 4 alcohols, 5 alkenes, and 2 furans were tentatively identified by comparing with the in-line database and retention index (RI). Among them, 24 volatiles were accurately identified by the authentic standards (Table 1). Some of the volatiles were similar to the volatiles produced by the germinated brown rice and bean sprout in previous studies [2,16,17], which showed the accuracy of volatile identification in our experiment.

**Figure 2:**
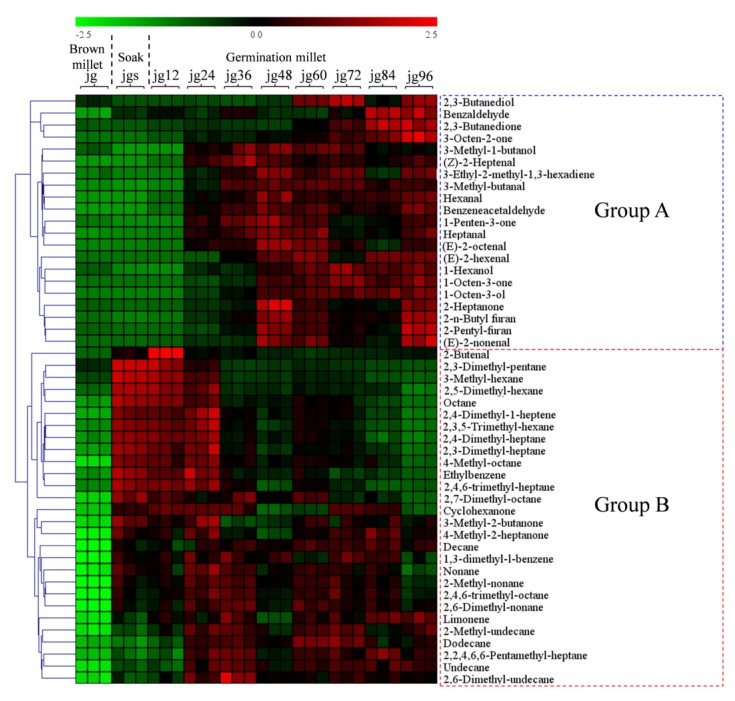
A heatmap of the contents of volatiles during germination of foxtail millet. The data were auto-scaled and clustered according to the Pearson correlation coefficients. Green colors indicated that the volatile level was less than its mean level during millet germination, while red colors indicated that the volatile level was higher than its mean level. Group A mainly contained ketones, aldehydes, alcohols, and furans, whereas group B mainly contained alkanes and alkenes. jg, jgs, and jg12–96 represented the raw brown millet, soaked brown millet, and germinated millet, respectively.

A heatmap was applied to show the variation content of the differential volatiles during millet germination. Green indicated that the volatile level was less than the mean level in all samples, whereas red indicated that the volatile level was higher than the mean level (Figure 2). The volatiles were clustered into two groups named group A and group B during millet germination. Interestingly, group A mainly containing ketones, aldehydes, alcohols, and furans began to accumulate at the 24-h germination stage, whereas group B mainly containing the alkanes and alkenes began to accumulate at the soaking stage. The variation patterns were agreed to a previous study that the volatiles were also increased in germinated brown rice [2]. The volatiles during millet germination are produced from the oxidation of the fatty acid [18]. At the soaking stage, the main volatile compounds were alkanes and alkenes. The reason might be due to the anaerobic environment of the soaking stage because both the alkanes and alkenes are do not have the oxygen atom. The alkanes and alkenes have gasoline-like odors with high thresholds. Therefore, these compounds might not be contributors to the smells of the germinated millet. After 24-h germination, the millet had a stronger odor than the soaked millet, which was due to the increase of the volatiles of ketones, aldehydes, alcohols, and furans. These volatiles have more diverse odors than alkanes and alkenes and are mainly produced from unsaturated fatty acids.

### 2.2. Variations of the Fatty Acid during Germination of the Foxtail Millet

**Figure 3:**
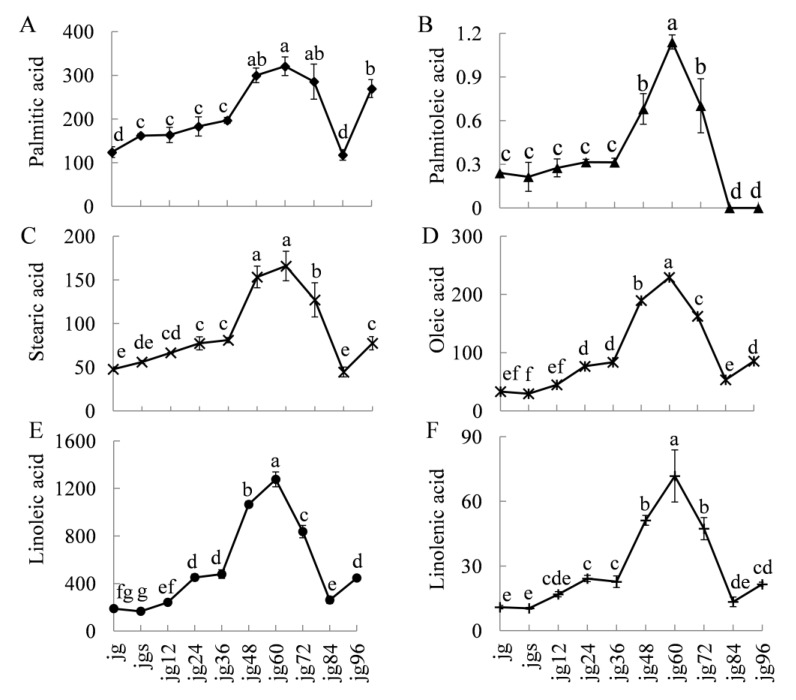
Variations in the contents of the fatty acids (mg kg^−1^) during millet germination. The significance of the differences (different letters) was determined at *p* < 0.05. Data were shown as the mean ± SD values (*n* = 3). jg, jgs, and jg 12–96 represented raw brown millet, soaked brown millet, and germinated millet for 12, 24, 36, 48, 60, 72, 84, and 96 h, respectively.

To investigate the relationship between the volatiles and fatty acids, six fatty acids were determined during the foxtail millet germination. Among them, palmitic acid, stearic acid, oleic acid, linoleic acid, and linolenic acid are the main compositions of the foxtail millet [19,20]. The contents of all the fatty acids (except palmitic acid) changed non-significantly after soaking, whereas they dramatically increased after 36-h germination and reached the maximum at 60-h germination (Figure 3). The same variation trend also takes place in the germination of peanut [4] and flaxseed [9]. The increase of the fatty acid might be due to the hydrolysis of the glyceride. The activity of lipase was increased during the seed germination (Figure S1), and results in the glyceride decreased [9]. However, after 60-h germination, the contents of the fatty acids began to decrease. The decrease of the fatty acid might be due to the β-oxidation, which can provide adenosine triphosphate (ATP) for millet germination.

### 2.3. Relationships between the Volatiles and Fatty Acid during Millet Germination

The increase of fatty acids may promote the increase of the volatiles. To further investigate the correlation of the volatiles and fatty acids during millet germination, a model experiment, which added the fatty acids (palmitic acid, palmitoleic acid, stearic acid, oleic acid, linoleic acid, and linolenic acid) to the crude enzymes, was designed to analyze the different volatiles based on a previous method with modifications [10].

Firstly, the volatiles produced by non-enzyme reactions must be excluded because the standard of unsaturated fatty acid is unstable during its storage and easily autooxidized. Treatment 1, which added the fatty acids to the phosphate buffer with heating 24 h at 37 °C, was used to estimate whether the volatiles were produced by the autooxidation of the fatty acids, or originally presented in the standard of fatty acid. Three fatty acids, linoleic acid, oleic acid, and palmitoleic acid, could be detected the volatiles in treatment 1 (Figure S2). It meant other fatty acids (palmitic acid, stearic acid, and linolenic acid) were stable during 24 h water bath. However, linolenic acid is also easily autooxidized. Thus, the volatiles detected in treatment 1 might be due to the autooxidation during long-time storage of the linoleic acid, oleic acid, and palmitoleic acid rather than 24 h water bath.

In the treatment 1 of linoleic acid, 1-hexanol, 2-pentyl-furan, and 3-octen-2-one were detected and were higher in contents than the treatment 2 and 3, respectively. It meant that the three volatiles originally existed in the linoleic acid rather than the enzymatic oxidation. In contrast, in palmitoleic acid, heptanal, (*E*)-2-octenal, and (*E*)-2-nonenal in treatment 1 were higher than those in treatment 2 but less than those in treatment 3. This indicated that the three volatiles not only existed in palmitoleic acid but also could be generated by enzymatic oxidation. In oleic acid, (*E*)-2-octenal in treatment 2 was higher in content than treatment 1 and 3. It meant something could produce the (*E*)-2-octenal in crude enzymes while a high concentration of oleic acid could inhibit its produce.

Higher volatiles in treatment 3 than treatment 2 indicated that these volatiles were produced by the corresponding fatty acid. Treatment 3 was performed by adding the fatty acids to the crude enzymes while treatment 2 was the crude enzyme only. After 24 h reaction at 37 °C, only trace of volatiles could be detected from palmitic acid and stearic acid in treatment 3 compared to treatment 2 (Figure S3). This phenomenon might be due to the inhibition effect of the palmitic acid and stearic acid on the generation of the volatiles. Meanwhile, the results showed that the saturated fatty acid did not produce the volatiles during the foxtail millet germination.

**Figure 4:**
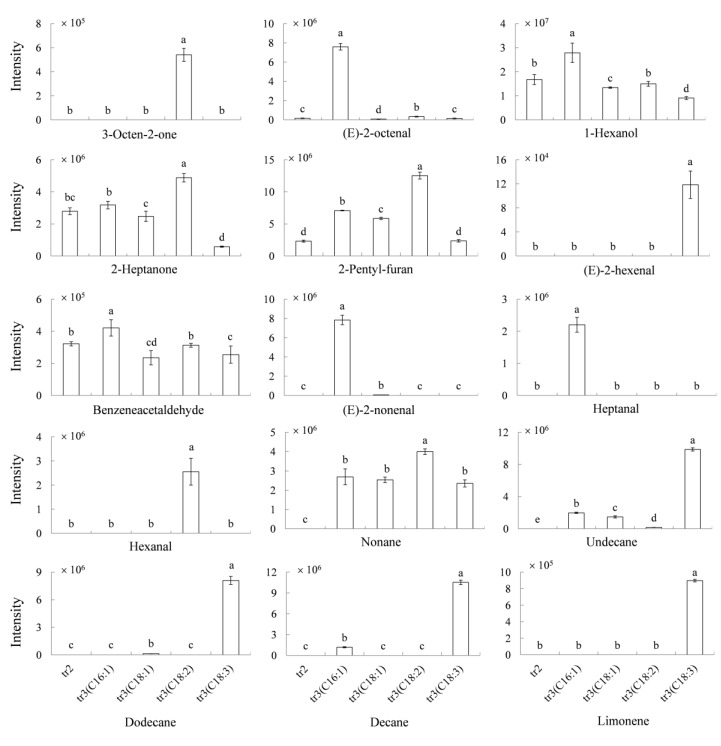
Model experiment to explain the generation of the volatiles from palmitoleic acid, oleic acid, linoleic acid, and linolenic acid. tr2 and tr3 represented treatment 2 and treatment 3, respectively. C16:1, C18:1, C18:2, and C18:3 represented palmitoleic acid, oleic acid, linoleic acid, and linolenic acid, respectively. The significance of the differences (different letters) was determined at *p* < 0.05. Data were shown as the mean ± SD values (*n* = 3).

In contrast, most volatiles were produced by unsaturated fatty acids (Figure 4). Six volatiles (2-heptanone, nonane, 2-pentyl-furan, 3-octen-2-one, hexanal, (*E*)-2-octenal) were significantly higher in linoleic acid in treatment 3 than in treatment 2, which indicated these volatiles could be produced by the linoleic acid. However, the formation of 2-pentyl-furan and 3-octen-2-one had been proved coming from the standard of linoleic acid according to the description above. Three volatiles, 1-octen-3-ol, benzeneacetaldehyde and 1-hexanol, were almost equally between the treatment 2 and treatment 3, which meant these volatiles did not come from the enzymatic oxidation of linoleic acid. In contrast, two volatiles (3-methyl-1-butanol and 2,3-butanediol) were at higher levels in treatment 2 than the treatment 3 of linoleic acid (Figure S4). The reason might be due to the inhibition effect of hydroperoxide on the generation of these two volatiles [21]. Similarly, 1-hexanol, heptanal, 2-pentyl-furan, (*E*)-nonenal, benzeneacetaldehyde, (*E*)-2-octenal, and undecane could be produced from the palmitoleic acid. (*E*)-nonenal, 2-pentyl-furan, undecane, and dodecane could be produced from the oleic acid. Nonane, decane, limonene, undecane, dodecane, and (*E*)-2-hexenal could be produced from the linolenic acid (Figure 4). The results also agreed to the previous study that hexanal, (*E*)-2-octenal, and 2-pentyl-furan are derived from linoleic acid, and *(E)*-2-hexenal is produced by the linolenic acid [10,22,23]. However, a previous study shows that the heptanal and 2-heptanone are produced from oleic acid [24], which is different from our results that these compounds were produced from palmitoleic acid. As the aldehydes, ketones, and alcohols present odors, and they are mainly produced by the palmitoleic acid, oleic acid, and linoleic acid, choosing the cultivar containing a low level of these fatty acids for germination might weak the bad odor of the germinated millet.

**Figure 5:**
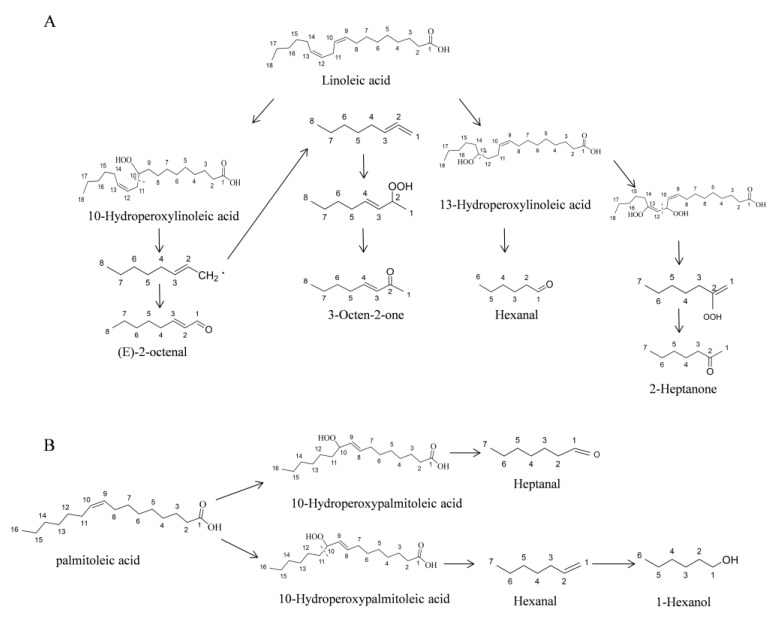
Proposed formation pathways of the volatiles produced from linoleic acid (**A**) and palmitoleic acid (**B**).

The hydroperoxides of unsaturated fatty acids were important intermediates to the formation of the volatiles. The hydroperoxide is formed through introducing dioxygen to the unsaturated fatty acids by lipoxygenase, and the 9- or 13-hydroperoxides are the most common compounds [10]. However, other hydroperoxides such as 8-, 10-, 12-, and 14-hydroperoxide also can be detected in minor components [25]. The cleavage position of the hydroperoxide decides the formation of the compounds. Two kinds of cleavage mechanism, heterolytic and homolytic, are involved in the degradation of the hydroperoxide [26]. The heterolytic is the cleavage between the carbon bearing the hydroperoxide group and the unsaturated carbon, and the formation of hexanal, 2-heptanone, hepatanal, and *(E)*-hexenal were proposed belonging to this kind of cleavage mode according to the previous descriptions (Figure 5) [26,27]. The formation of *(E)*-2-octenal, 3-octen-2-one, and 1-hexanol belonged to the hemolytic, which is the cleavage of the hydroperoxide between the carbon bearing the hydroperoxide group and the saturated carbon [26].

It was noteworthy that 2-pentyl-furan was increased in the palmitoleic acid, oleic acid, and linoleic acid in treatment 3. The 2-pentyl-furan has been widely detected in the oils with fruity, green, and earthy odor [28,29]. Previous studies report its formation is attributed to the oxidation of the unsaturated aldehydes that usually generated from the polyunsaturated fatty acid [28,30]. However, in our research, the 2-pentyl-furan also could be detected from monounsaturated fatty acids (palmitoleic acid and oleic acid) as well as the monounsaturated aldehydes (*(E)*-nonenal and *(E)*-2-octenal). Thus, their formations from the monounsaturated fatty acid need further investigations. Moreover, the alkanes and alkenes were also increased in treatment 3, especially in the linoleinic acid. Though these compounds are almost no contribution to the aroma of the germinated foxtail millet, they are commonly produced by the fatty acids [2,31]. However, to our best knowledge, the formation of the alkanes and alkenes from the unsaturated fatty acid is still unclear and need a further investigation.

## 3. Materials and Methods

### 3.1. Chemicals

Fatty acid methyl esters of palmitic acid (C16:0), palmitoleic acid (C16:1), stearic acid (C18:0), oleic acid (C18:1), linoleic acid (C18:2) and linolenic acid (C18:3), C_3_-C_25_ of n-alkanes, 2,3-butanedione, hexanal, *(E)*-2-hexenal, 1-octen-3-ol, 1-octen-3-one, 2-heptanone, *(E)*-2-octenal, and 1-hexanol were purchased from Sigma (St. Louis, MO, USA). 3-Octen-2-one, 2-n-butyl furan, heptanal, and *(E)*-2-nonenal were purchased from TCI (Tokyo, Japan). Benzeneacetaldehyde, 2,3-butanediol, 3-methyl-2-butanone, and benzaldehyde were obtained from RHAWN reagent corporation (Shanghai, China). 1-Penten-3-one and 3-methyl-1-butanol were purchased from Aladdin reagent corporation (Shanghai, China). 2-Pentyl-furan was purchased from J&K Scientific Ltd. (Beijing, China). Heptenal was purchased from Accustandard (New Haven, CT, USA). Diethyl ether, n-hexane, and petroleum ether were purchased from Yongda chemical reagent co. Ltd. (Tianjin, China). Boron trifluoride-methanol solution (14% BF3·methanol) was purchased from CNW technologies (Dusseldorf, Germany). Milli-Q water was used in this study (Millipore, Billerica, MA, USA).

### 3.2. Sample of Foxtail Millet

The seed of the foxtail millet (cultivar of Jigu 19) was harvested in Gaocheng district, Shijiazhuang City, Hebei, China, on 18 September 2018. The brown millet was made by removing the bran of the millet with the germ being reserved using a rice mill (SY88-TH, Ssang Yong Machinery industry co. Ltd., Incheon, Korea). The method of brown millet germination was according to a previous study with some modifications [7]. Briefly, 500 g brown millet was soaked in water for 12 h to absorb enough water, and germinated in a biochemical incubator (SPX, Linmaokeji co. Ltd., Beijing, China) at 25 °C with 60% humidity for 96 h. Every 12 h, the germinated millet was sampled. Finally, the total samples were divided into three batches: raw brown millet, soaked millet, and germinated millet for 12, 24, 36, 48, 60, 72, 84, and 96 h. All samples were immediately frozen with liquid nitrogen and then freeze-dried using vacuum freeze-drying equipment (D37520, Martin Christ, Osterode, Lower Saxony, Germany). The obtained samples were pulverized into powders with a pulverizer (IKA, Staufen, Germany) and stored at −20 °C for future use.

### 3.3. Determination of Volatiles Using HS-SPME-GC×GC-TOF/MS

The volatiles were measured using headspace solid-phase microextraction (HS-SPME)-GC×GC-TOF/MS. Before volatile extraction, the SPME fiber was preconditioned at 250 °C for 1 h in the injection port of the GC×GC-TOF/MS to activate the absorption ability. The SPME of the volatiles of the millet was performed according to the method described in a previous study [32]. Briefly, 1 g of sample was infused with 0.1 mL of pure water in a 20 mL vial with a screw cap, and the solution was heated in a water bath at 50 °C for 30 min. Accompanying with heating, the volatiles were absorbed by HS-SPME with a 50/30 μm divinylbenzene/carboxen/polydimethylsiloxane (DVB/CAR/PDMS) fiber (Supelco, Inc., Bellefonte, PA, USA) in an automatic headspace sampling system (CTC analytics, Zwingen, Switzerland). The SPME fiber was then introduced into the injector port of GC and kept for 5 min to allow thermal desorption of the volatiles. Each sample was tested in triplicate.

The parameters of the GC×GC-TOF/MS instrument (LECO Corporation, St. Joseph, MI, USA) were according to a previous study with slight modifications [33]. In brief, the injector temperature was 250 °C with splitless injection. A DB-5MS column (30m × 0.25mm i.d., 0.25 μm film thickness, Agilent Technologies, Santa Clara, CA, USA) was used for the first-dimension analysis. The initial temperature of the column oven was 35 °C for 2 min, and then it was heated at 8 °C/min to 220 °C and held for 5 min. The second-dimensional column was DB-17ht (1.9 m × 0.1 mm i.d., 0.10 μm film thickness, Agilent, Santa Clara, CA, USA) and + 10 °C above the first-dimension column. The flow rate of carrier gas (Helium, purity > 99.999%) was 1 mL/min. The ionization voltage was 70 eV (EI), and the ion source temperature was 230 °C. The interface temperature was 250 °C. The electron multiplier was at 1800 V and the mass scan range was *m*/*z* 33–600. After sample injection, the acquisition delay was 180 s.

### 3.4. Determination of Fatty Acid Using GC-FID

Fatty acid extraction and derivatization were conducted by a previous study with some modifications [34]. Briefly, 1 g of millet powder with 30 mL of diethyl ether and petroleum ether (1:1, *v*/*v*) were sufficiently mixed in a 50 mL centrifuge tube and shook for 2 h at 250 r/min using a shaker (MAXQ 4000, Thermo Scientific, Santa Clara, CA, USA). Then, the mixture was centrifuged at 4000 r/min for 10 min (centrifuge 3SR+, Thermo Scientific, CA, USA), and the supernatants were dried in a rotavapor at 35 °C (Buchi R215, Flawil, Switzerland). The dried residuals were re-dissolved in 1 mL of 14% BF_3_-methanol solution and water-bathed at 80 °C for 2 min. The derivative reaction was terminated by placing it in the ice, and the fatty acid methyl ester was extracted by 2 mL n-hexane.

The fatty acid methyl esters of palmitic acid, palmitoleic acid, stearic acid, oleic acid, linoleic acid, and linolenic acid were determined by the method described in a previous study with slight modifications [34]. Briefly, it was performed with a GC-flame ionization detector (FID) (7820A, Agilent Technologies, Santa Clara, CA, USA). The injector temperature was 260 °C with splitless. A HP-INNOWAX column (30 m × 0.25 mm i.d., 0.25 μm film thickness, Agilent Technologies, Santa Clara, CA, USA) was used for analysis. The initial temperature of the column oven was 50 °C, and then it was heated at 10 °C/min to 150 °C and held for 3 min. The oven was then heated at 4 °C/min to 205 °C for 1 min and at the rate of 8 °C/min to the final temperature of 235 °C for 2 min. The carrier gas (Nitrogen, purity > 99.999%) was at a constant flow rate of 1 mL/min. The detector temperature was 260 °C. The airflow and hydrogen flow were 400 and 30 mL/min, respectively. Finally, a total of six external standard calibration curves were generated to quantify fatty acids (Table S1).

### 3.5. Determination of the Activity of Lipase

The determination of the activity of lipase was according to a previous study with some modifications [35]. Briefly, 1.00 g brown millet powder was infused with 5 mL Tris-HCl (pH 7.5) for 1 h at 4 °C for extracting the crude enzymes, and then the mixture was centrifuged at 8000 r/min for 10 min (centrifuge 3SR+, Thermo Scientific, CA, USA). 1.5 mL supernatant was mixed with 1 mL KCl (0.5 mol/L), 1 mL CaCl_2_ (5 mmol/L), 0.5 mL Tris-HCl, and 1 mL glycerol triacetate, and water-bathed at 37 °C for 1 h. Acetic acid would be produced through the catalysis of glycerol triacetate by lipase and was titrated with 0.05 mol/L NaOH solution using the phenolphthalein as indicator. 1 U of lipase was defined as the amounts of 0.01 mL NaOH (0.05 mol/L) was consumed by 1 g brown millet powder at 1 min.

### 3.6. Model Experiments to Explain the Relationship between the Volatiles and the Fatty Acids with Crude Enzymes

The crude enzymes were prepared according to the method described in a previous study with slight modifications [10]. Ten grams of each sample were mixed with 300 mL phosphate buffer (0.2 mol/L, pH 6.0 added 0.3 mL Triton X-100). The mixture was homogenized for 30 min (homogenizer, IKA T-25, Staufen, Germany), and the homogenate was centrifuged at 8000 r/min for 10 min (centrifuge 3SR+, Thermo Scientific, CA, USA). The supernatant containing the crude enzymes was stored at 4 °C for experiment use.

**Figure 6:**
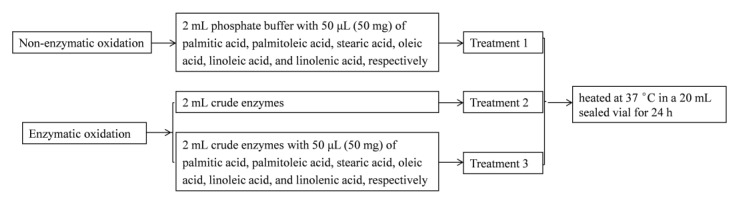
Experimental design to elucidate the relationships between the volatiles and fatty acids.

Palmitic acid, palmitoleic acid, stearic acid, oleic acid, linoleic acid, and linolenic acid were used as precursors of volatiles in the model experiments. The model experiment was performed in two parts in a 20 mL sealed vial (Figure 6). One part was used to exclude the volatiles originally existed in the fatty acid or produced by autooxidation of each fatty acid during 24 h water bath (treatment 1), and the other part was the enzymatic oxidation of each fatty acid (treatment 2 and 3). All treatments were water-bathed at 37 °C for 24 h and then stored at −20 °C for detecting the volatiles.

The volatile of the model experiment was detected using HS-SPME-GC-MS (7890A GC, 5975C MS, Agilent Technologies, Santa Clara, CA, USA). The parameters were the same as the method of HS-SPME-GC×GC-TOF/MS above, but only a DB-5MS column was used for analysis.

### 3.7. Data Treatment and Statistical Analysis

The data obtained by GC×GC-TOF/MS were processed using the LECO ChromaTOF software. The peak width of the 1st D and 2nd D was set to 25 and 0.1, respectively. The minimum S/N was set to 20 with mass range of 33–600 *m*/*z*, and the minimum similarity, reverse, and probability were set to 750, 750, and 1000, respectively. Total processing time was set to 33.875 min. The chemical structure was tentatively identified through comparison with database of “mainlib”, “replib”, and “nist_ri”. After data preprocessing, the statistical comparison function in the ChromaTOF software was used to compare and integrate the obtained data to establish a peak list.

The principal component analysis (PCA) and partial least squares-discriminate analysis (PLS-DA) were performed with Simca-P software (version 11.5, Umetrics AB, Umeå, Sweden) with Pareto-scaling (mean-centered and divided by the square root of SD). The PLS-DA was carried out by labeling the sample of jg as class 1, and other samples as class 2. The ions with variable importance in projection (VIP > 1) were used to screen the differential volatiles. A cluster analysis was conducted with a Pearson correlation after auto-scaling using MultiExperiment Viewer 4.9.0 software. One-way analysis of variance (ANOVA) was employed to analyze the significant difference in the contents of the fatty acids during millet germination using Duncan’s multiple range tests at the level of *p* < 0.05 using the SPSS 21.0 software (IBM, Worcester, MA, USA).

## 4. Conclusions

Forty-nine volatiles containing 8 ketones, 10 aldehydes, 20 alkanes, 4 alcohols, 5 alkenes, and 2 furans were tentatively identified during germination of the foxtail millet and divided into two groups according to the period of germination. The alkanes and alkenes were mainly produced at the soaking stage, whereas the aldehydes, alcohols, and ketones were mainly produced at the germinating stage. The relationship between the volatiles and fatty acids was also analyzed by model experiments, and 17 volatiles were detected. The palmitic acid and stearic acid had no contribution to the formation of the volatiles, whereas the unsaturated fatty acid played important roles in the formation of volatiles. Among the unsaturated fatty acids, palmitoleic acid and linoleic acid produced most aldehydes, alcohols, and ketones, while linolenic acid produced the most alkanes and alkenes.

## Figures and Tables

**Table 1:** Differential volatile compounds putatively identified during germination of the foxtail millet.

ID	Volatile Compound	1st Rt	2nd Rt	RIcal ^a^	RIref ^b^	Probability	Odor Description ^c^
Ketones							
1	2,3-Butanedione ^d^	3.40	1.79	566	595	1225	fruity
2	1-Penten-3-one ^d^	4.60	2.07	644	681	7650	pungent
3	3-Methyl-2-butanone ^d^	4.60	2.01	644	657	1443	camphor
4	2-Heptanone ^d^	8.73	2.18	880	891	8707	fruity, floral
5	Cyclohexanone	8.87	2.77	887	894	7841	minty acetone
6	4-Methyl-2-heptanone	9.80	2.12	932	943	8771	NF
7	1-Octen-3-one ^d^	10.67	2.25	972	979	6443	mushroom, earthy
8	3-Octen-2-one ^d^	11.93	2.34	1035	1033	4690	nutty, fruity
Aldehydes							
9	2-Butenal	4.07	2.11	610	629	3657	flower
10	3-Methyl-butanal	4.73	2.04	653	652	1162	peach
11	Hexanal ^d^	6.73	2.19	776	800	8959	grass
12	(*E*)-2-hexenal ^d^	8.00	2.33	844	854	5388	green banana, cheesy
13	Heptanal ^d^	9.00	2.20	894	901	8838	herbal
14	(*Z*)-2-Heptenal	10.20	2.36	950	958	4277	pungent, vegetable
15	Benzaldehyde ^d^	10.33	2.94	957	962	8697	bitter almond
16	Benzeneacetaldehyde ^d^	12.07	3.00	1042	1045	8017	green sweet, floral
17	(*E*)-2-octenal ^d^	12.33	2.35	1056	1060	7333	fresh cucumber
18	(*E*)-2-nonenal ^d^	14.33	2.31	1161	1162	6268	green cucumber
Alkanes							
19	2,3-Dimethyl-pentane	4.73	1.60	652	672	2381	NF
20	3-Methyl-hexane	5.33	1.61	691	676	859	NF
21	2,5-Dimethyl-hexane	6.00	1.65	733	729	967	NF
22	Octane ^d^	6.73	1.68	776	800	5265	gasoline
23	2,3,5-Trimethyl-hexane	7.07	1.67	796	812	2323	NF
24	2,4-Dimethyl-heptane	7.20	1.68	803	821	1831	NF
25	2,3-Dimethyl-heptane	7.93	1.70	840	855	3634	NF
26	4-Methyl-octane	8.13	1.70	850	863	5801	NF
27	2,4,6-trimethyl-heptane	8.33	1.68	860	870	2161	NF
28	Nonane ^d^	8.93	1.71	890	900	3280	gasoline
29	2,7-Dimethyl-octane	9.53	1.69	919	928	5752	NF
30	2-Methyl-nonane	10.33	1.71	9569	964	3699	NF
31	2,4,6-trimethyl-octane	10.60	1.68	969	959	1045	NF
32	2,2,4,6,6-Pentamethyl-heptane	10.93	1.70	9849	991	6719	NF
33	Decane ^d^	11.07	1.76	991	1000	5144	alkane
34	2,6-Dimethyl-nonane	11.53	1.72	1014	1018	1208	NF
35	Undecane ^d^	13.13	1.74	1097	1100	1821	alkane
36	2-Methyl-undecane	14.33	1.76	1161	1164	4077	NF
37	Dodecane ^d^	15.00	1.79	1196	1200	3576	alkane
38	2,6-Dimethyl-undecane	15.27	1.76	1212	1210	2783	NF
Alcohols							
39	3-Methyl-1-butanol ^d^	6.07	2.05	736	736	1758	fusel oil, whiskey
40	2,3-Butanediol ^d^	6.53	2.38	765	788	9524	fruity, creamy
41	1-Hexanol ^d^	8.27	2.13	857	868	5363	ethereal fusel oil
42	1-Octen-3-ol ^d^	10.67	2.11	972	980	4162	mushroom, earthy
Alkenes							
43	2,4-Dimethyl-1-heptene	7.67	1.73	827	836	6350	NF
44	Ethylbenzene	8.13	2.23	850	855	4960	NF
45	1,3-dimethyl-l-benzene	8.33	2.19	860	866	3038	plastic
46	Limonene	11.73	2.06	1024	1030	6216	citrus
47	3-Ethyl-2-methyl-1,3-hexadiene	11.80	2.57	1028	1031	912	NF
Furans							
48	2-n-Butyl furan ^d^	8.80	2.04	884	893	9053	mild fruity wine
49	2-Pentyl-furan ^d^	10.93	2.08	985	993	8130	fruity, green, earthy

^a^ Retention index (RI) is calculated in this study. ^b^ RI is referred from the NIST database. ^c^ odor description is adopted from the database (http://www.thegoodscentscompany.com/index.html). ^d^ confirmed by authentic standards. NF means not found in references and databases.

## Supplementary Materials

The following are available online, Figure S1: Variation pattern of the lipase activity during foxtail millet germination. Figure S2: Volatiles are produced by the autoxidation of palmitoleic acid, linoleic acid, and oleic acid. Figure S3: Volatiles are produced by the enzymatic oxidation of palmitic acid and stearic acid. Figure S4: 3-Methyl-butanol and 2,3-Butanediol are not produced by the enzymatic oxidation of the palmitoleic acid, oleic acid, linoleic acid, and linolenic acid. Table S1: Calibration curves for quantification of the fatty acids.

## References

[1] Li X., Kim Y.B., Uddin M.R., Lee S., Kim S.J., Park S.U. (2013). Influence of light on the free amino acid content and γ-aminobutyric acid synthesis in *Brassica juncea* seedlings. J. Agric. Food Chem..

[2] Xia Q., Mei J., Yu W., Li Y. (2017). High hydrostatic pressure treatments enhance volatile components of pre-germinated brown rice revealed by aromatic fingerprinting based on HS-SPME/GC–MS and chemometric methods. Eur. Food Res. Technol..

[3] Coulibaly A., Chen J. (2011). Evolution of energetic compounds, antioxidant capacity, some vitamins and minerals, phytase and amylase activity during the germination of foxtail millet. Am. J. Food Technol..

[4] Chiou R.Y.Y., And K.L.K., Chen W.L. (1994). Compositional characterization of peanut kernels after subjection to various germination times. J. Agric. Food Chem..

[5] Guardado-Felix D., Serna-Saldivar S.O., Cuevas-Rodriguez E.O., Jacobo-Velazquez D.A., Gutierrez-Uribe J.A. (2017). Effect of sodium selenite on isoflavonoid contents and antioxidant capacity of chickpea (*Cicer arietinum* L.) sprouts. Food Chem..

[6] Sharma S., Saxena D.C., Riar C.S. (2015). Antioxidant activity, total phenolics, flavonoids and antinutritional characteristics of germinated foxtail millet (*Setaria italica*). Cogent Food Agric..

[7] Li X., Hao J., Liu X., Liu H., Ning Y., Cheng R., Tan B., Jia Y. (2015). Effect of the treatment by slightly acidic electrolyzed water on the accumulation of γ-aminobutyric acid in germinated brown millet. Food Chem..

[8] Bai Q., Fan G., Gu Z., Cao X., Gu F. (2008). Effects of culture conditions on γ-aminobutyric acid accumulation during germination of foxtail millet (*Setaria italica* L.). Eur. Food Res. Technol..

[9] Wanasundara P.K.J.P.D., Wanasundara U.N., Shahidi F. (1999). Changes in flax (*Linum usitatissimum* L.) seed lipids during germination. J. Am. Oil Chem. Soc..

[10] Ghita Studsgaard N., Lone Melchior L., Leif P. (2004). Formation of volatile compounds in model experiments with crude leek (*Allium ampeloprasum* Var. *Lancelot*) enzyme extract and linoleic acid or linolenic acid. J. Agric. Food Chem..

[11] Matsui K., Toyota H., Kajiwara T., Kakuno T., Hatanaka A. (1991). Fatty acid hydroperoxide cleaving enzyme, hydroperoxide lyase, from tea leaves. Phytochemistry.

[12] Ohta H., Ida S., Mikami B., Morita Y. (1986). Changes in lipoxygenase components of rice seedlings during germination. Plant. Cell Physiol..

[13] Hisao K., Hidetoshi K., Hirotaka K., Masachika T. (2005). Characterization of 9-fatty acid hydroperoxide lyase-like activity in germinating barley seeds that transforms 9(S)-hydroperoxy-10*(E)*,12(Z)-octadecadienoic acid into *(E)*-nonenal. Biosci. Biotechnol. Biochem..

[14] Salas J.J., García-Gonzalez D.L., Ramón A. (2006). Volatile compound biosynthesis by green leaves from an *Arabidopsis thaliana* hydroperoxide lyase knockout mutant. J. Agric. Food Chem..

[15] Qin G., Tao S., Zhang H., Huang W., Wu J., Xu Y., Zhang S. (2014). Evolution of the aroma volatiles of pear fruits supplemented with fatty acid metabolic precursors. Molecules.

[16] Liu K., Zhao S., Li Y., Chen F. (2018). Analysis of volatiles in brown rice, germinated brown rice, and selenized germinated brown rice during storage at different vacuum levels. J. Sci. Food Agric..

[17] Zuo Y., Ju S., Liu L.P., Li Y., Xie H. (2011). Analysis of volatile compounds in bean sprout by simultaneous distillation extraction and gas chromatography-mass spectrometry. Adv. Mater. Res..

[18] Horiuchi M., Umano K., Shibamoto T. (1998). Analysis of volatile compounds formed from fish oil heated with cysteine and trimethylamine oxide. J. Agric. Food Chem..

[19] Antony U., Sripriya G., Chandra T.S. (1996). The effect of fermentation on the primary nutrients in foxtail millet (*Setaria italica*). Food Chem..

[20] Zhang A., Liu X., Wang G., Wang H., Liu J., Zhao W., Zhang Y. (2015). Crude fat content and fatty acid profile and their correlations in foxtail millet. Cereal Chem..

[21] Sekiya J., Kajiwara T., Hatanaka A. (1979). Volatile C6-aldehyde formation via hydroperoxides from C18-unsaturated fatty acids in etiolated alfalfa and cucumber seedlings. J. Agric. Chem. Soc. Jpn..

[22] Zhang Y., Yang N., Fray R.G., Fisk I., Liu C., Li H., Han Y. (2018). Characterization of volatile aroma compounds after in-vial cooking of foxtail millet porridge with gas chromatography-mass spectrometry. J. Cereal Sci..

[23] Sekiya J., Kamiuchi H., Hatanaka A. (1982). Lipoxygenase, hydroperoxide lyase and volatile C6-aldehyde formation from C18-fatty acids during development of *Phaseolus vulgaris* L.. Plant. Cell Physiol..

[24] Monsoor M.A., Proctor A. (2010). Volatile component analysis of commercially milled head and broken rice. J. Food Sci..

[25] Haslbeck F., Grosch W. (2010). HPLC analysis of all positional isomers of the monohydroperoxides formed by soybean lipoxygenases during oxidation of linoleic acid. J. Food Biochem..

[26] Combet E., Eastwood D.C., Burton K.S., Combet E., Henderson J., Henderson J., Combet E. (2006). Eight-carbon volatiles in mushrooms and fungi: Properties, analysis, and biosynthesis. Mycoscience.

[27] Schieberle P., Grosch W. (1981). Model experiments about the formation of volatile carbonyl compounds. J. Am. Oil Chem. Soc..

[28] Min D.B., Callison A.L., Lee H.O. (2010). Singlet oxygen oxidation for 2-pentylfuran and 2-pentenyfuran formation in soybean oil. J. Food Sci..

[29] Krishnamurthy R.G., Smouse T.H., Mookherjee B.D., Reddy B.R., Chang S.S. (2010). Identification of 2-pentyl furan in fats and oils and its relationship to the reversion flavor of soybean oil. J. Food Sci..

[30] An A., Capucine B., Fien V.L., Bruno D.M., Norbert D.K. (2011). Amino acid catalysis of 2-alkylfuran formation from lipid oxidation-derived α,β-unsaturated aldehydes. J. Agric. Food Chem..

[31] Giogios I., Grigorakis K., Nengas I., Papasolomontos S., Papaioannou N., Alexis M.N. (2010). Fatty acid composition and volatile compounds of selected marine oils and meals. J. Sci. Food Agric..

[32] Klensporf D., Jelen’ H.H. (2005). Analysis of volatile aldehydes in oat flakes by SPME-GC/MS. Pol. J. Food Nutr. Sci..

[33] Zhao F., Liu J., Wang X., Li P., Zhang W., Zhang Q. (2013). Detection of adulteration of sesame and peanut oils via volatiles by GC × GC–TOF/MS coupled with principal components analysis and cluster analysis. Eur. J. Lipid Sci. Technol..

[34] Ivanova-Petropulos V., Mitrev S., Stafilov T., Markova N., Leitner E., Lankmayr E., Siegmund B. (2015). Characterisation of traditional Macedonian edible oils by their fatty acid composition and their volatile compounds. Eur. Food Res. Technol..

[35] Guo Y., Zhu X., Fang F., Hong X., Wu H., Chen D., Huang X. (2018). Immobilization of Enzymes on a Phospholipid Bionically Modified Polysulfone Gradient-Pore Membrane for the Enhanced Performance of Enzymatic Membrane Bioreactors. Molecules.

