# Supplementary material for: Variation Patterns of the Volatiles during Germination of the Foxtail Millet (Setaria Italic): The Relationship between the Volatiles and Fatty Acids in Model Experiments
Source: Molecules. 2020 Mar 9;25(5):1238. doi: 10.3390/molecules25051238 (PMC7179401; doi:10.3390/molecules25051238)
Supplement: Supplementary file 1 [file molecules-25-01238-s001.pdf]

Variation patterns of the volatiles during germination of the foxtail millet (*Setaria italic*): The relationship between the volatiles and fatty acids in model experiments

Pengliang Li[1, a], Yin Zhu[2, a], Shaohui Li[1], Aixia Zhang[1], Wei Zhao[1], Jiali Zhang[1], Qincao Chen[2], Sufen Ren[1], Jingke Liu[1, *], and Huijun Wang[1, *]

[a] **These authors contributed equally to this work.**

1 Institute of millet crops, Hebei Academy of Agriculture and Forestry Sciences, 162 Hengshan Street, Shijiazhuang, Hebei 050035, People's Republic of China

2 Key Laboratory of Tea Biology and Resources Utilization, Ministry of Agriculture, Tea Research Institute, Chinese Academy of Agricultural Sciences, 9 Meiling South Road, Hangzhou, Zhejiang 310008, People's Republic of China

* Correspondence: liujingke79@163.com; Tel.: +86-311-87670703 (Liu and Wang)

Table S1 Calibration curves for quantification of the fatty acids

Fatty acid methyl esters	Calibration curve	R^2	Linear range μg/mL
palmitic acid	y = 122318.9x + 81530	0.9999	1.64-246.3
palmitoleic acid	y = 136875.8x - 3022	0.9985	0.16-24.63
stearic acid	y = 125247.4x - 97267	0.9986	0.77-114.94
oleic acid	y = 134372.8x - 365317	0.9996	5.47-336.61
linoleic acid	y = 137537.5x - 48965	0.9985	0.38-57.5
linolenic acid	y = 135542.2x - 12588	0.9993	0.16-24.63

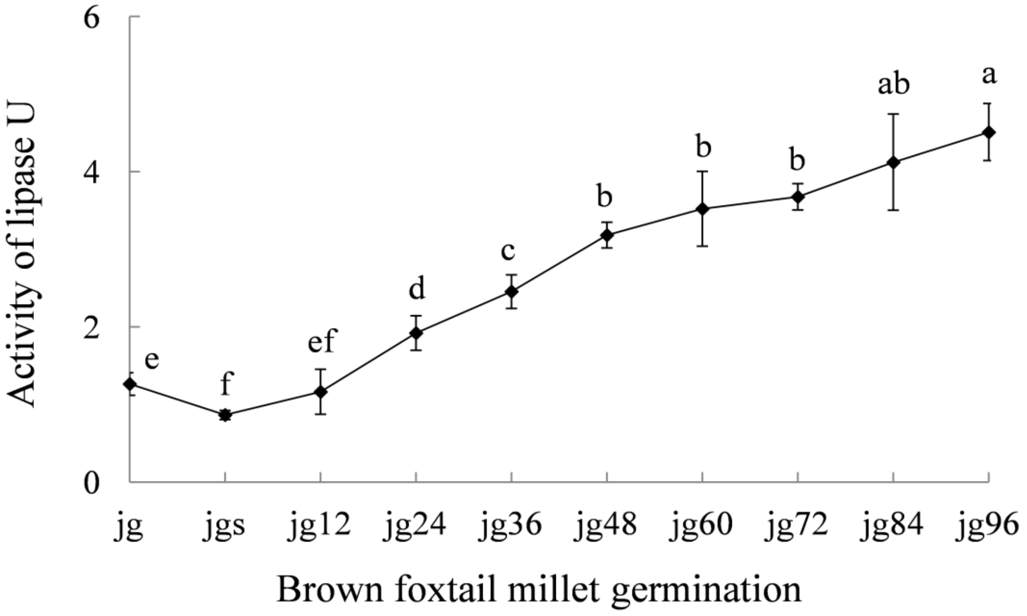

Figure S1. Variation pattern of the lipase activity during foxtail millet germination. jg, jgs, and jg12-96 represent the raw brown millet, soaked brown millet, and germinated millet, respectively. Different letter means significant different ($P < 0.05$) in data during germination of foxtail millet.

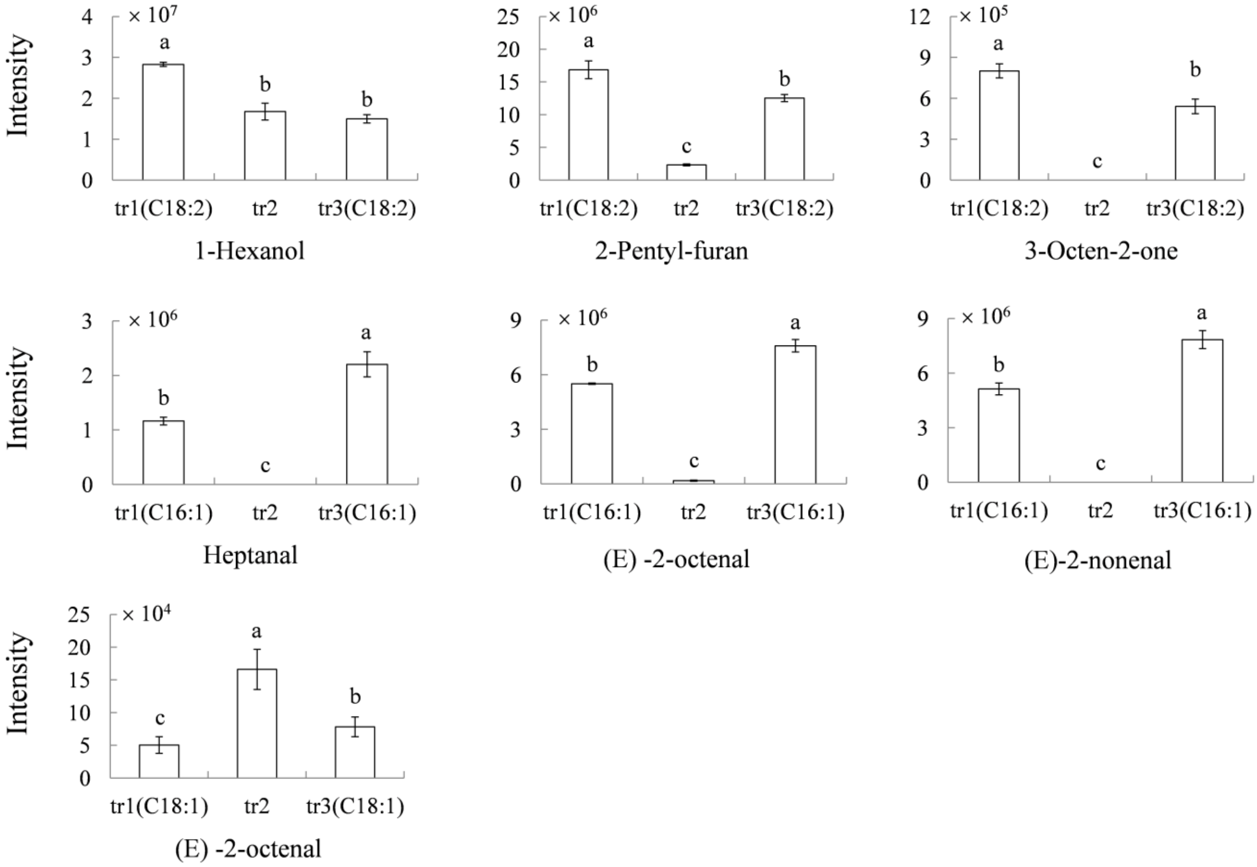

Figure S2. Volatiles are produced by the autoxidation of palmitoleic acid, linoleic acid, and oleic acid. tr1, tr2, and tr3 represent the treatment 1, treatment 2 and treatment 3, respectively. C16:1, C18:1, and C18:2 represent palmitoleic acid, oleic acid, and linoleic acid, respectively. The significance of the differences (different letters) is determined at $P < 0.05$. Data are the mean ± SD values (n = 3).

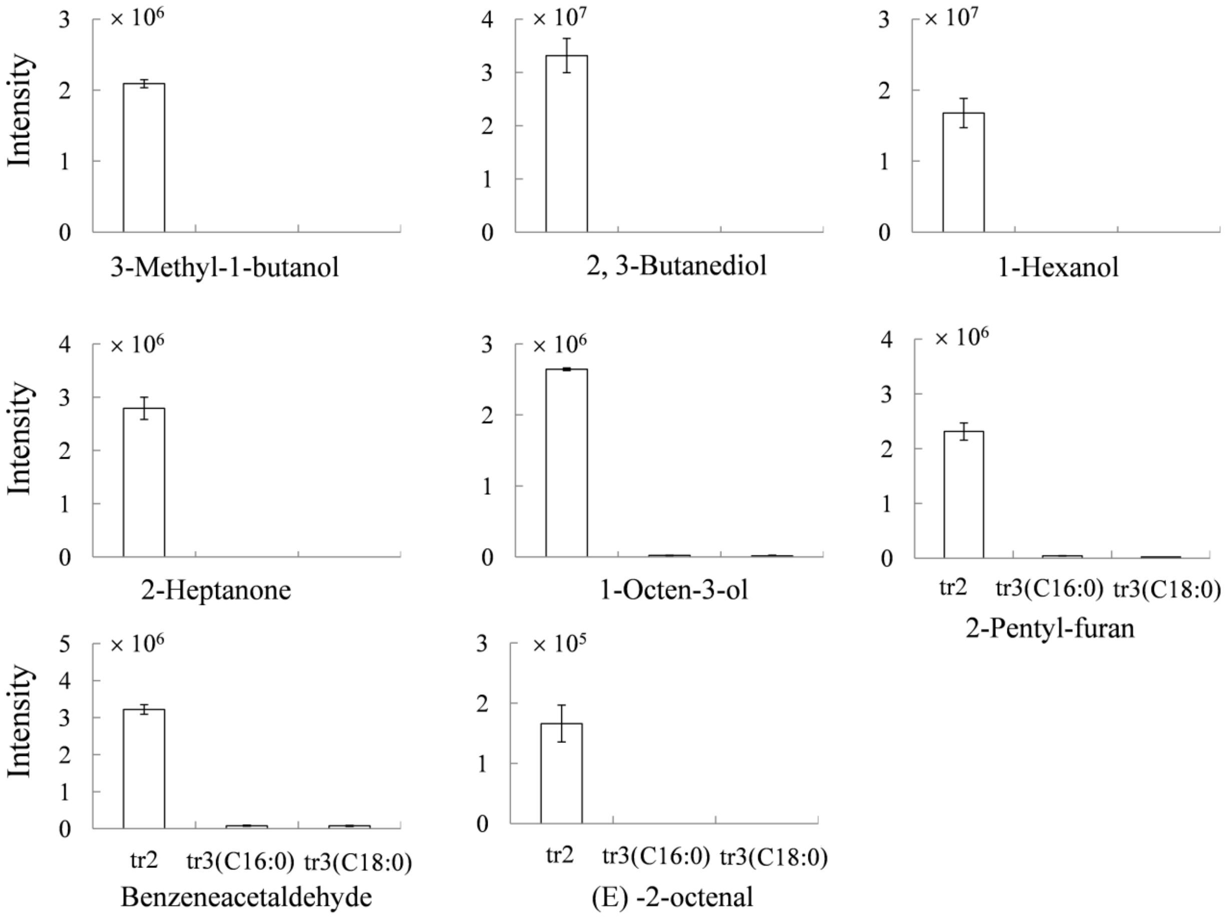

Figure S3. Volatiles are produced by the enzymatic oxidation of palmitic acid, and stearic acid. tr2 and tr3 represent the treatment 2 and treatment 3, respectively. C16:0 and C18:0 represent palmitic acid and stearic acid, respectively. The volatiles in treatment 3 are far less than in treatment 2. Data are the mean ± SD values (n = 3).

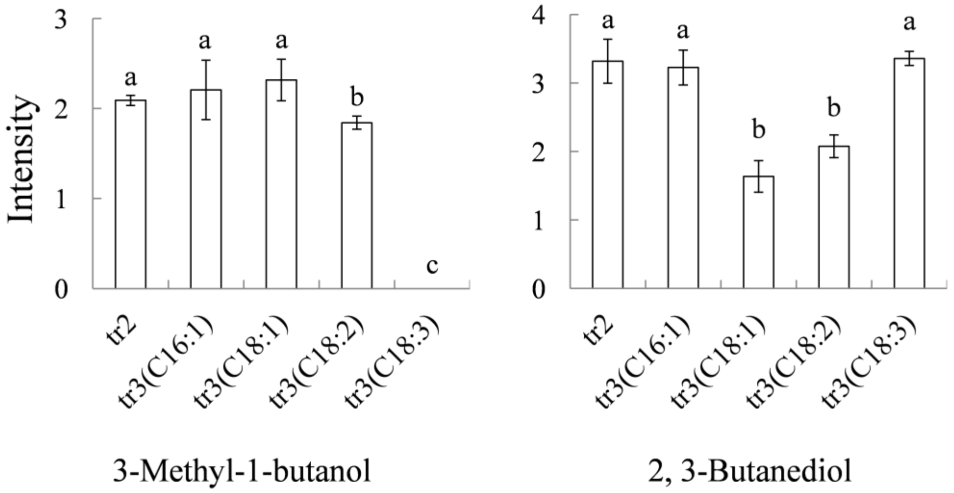

Figure S4. 3-Methyl-butanol and 2,3-Butanediol are not produced by the enzymatic oxidation of the palmitoleic acid, oleic acid, linoleic acid, and linolenic acid. tr2 and tr3 represent the treatment 2 and treatment 3, respectively. C16:1, C18:1, C18:2 and C18:3 represent palmitoleic acid, oleic acid, linoleic acid, and linolenic acid, respectively. The significance of the differences (different letters) is determined at $P < 0.05$. Data are the mean ± SD values (n = 3).
